# Engineering tandem VHHs to target different epitopes to enhance antibody‐dependent cell‐mediated cytotoxicity

**DOI:** 10.1002/2211-5463.70166

**Published:** 2025-11-20

**Authors:** Yuqiang Xu, Hao Jiang, Limin Chen, Fulai Zhou, Ying Jin, Mark L. Chiu

**Affiliations:** ^1^ Research & Development Department Tavotek Biotherapeutics Suzhou China; ^2^ Research & Development Tavotek Biotherapeutics Spring House PA USA

**Keywords:** ADCC, bispecific antibody, EGFR, Engineering VHH, single domain antibodies

## Abstract

Engineering antibodies to elicit antibody‐dependent cell‐mediated cytotoxicity (ADCC) can be used to eliminate target cells. Here, we described how single domain VHH arms on a bispecific antibody (BsAb) format can be engineered to modulate cell binding and ADCC activity. The BsAbs, comprising two anti‐epidermal growth factor receptor (EGFR) nanobodies, 7D12 and EGA1, were engineered onto a human IgG1 Fc domain in monovalent, bivalent, and tandem formats. While 7D12 had a stronger ADCC activity than EGA1, the tandem 7D12‐EGA1 mediated a significantly stronger ADCC activity without significant changes in cell binding in numerous cancer cell lines. In addition, we present how the molecular design of the tandem 7D12 and EGA1 nanobodies could cross‐link two different EGFR molecules to obtain stronger ADCC activity.

## Graphical abstract

Tandem VHH targeting distinct EGFR epitopes were engineered into a monovalent bispecific antibody (7D12‐EGA1‐Fc) with more potent ADCC without increasing affinity to EGFR. Structural modeling of 7D12‐EGA1‐Fc showed cross‐linking of separate EGFR domains to enhance CD16a engagement on NK cells. Such directed Fc clustering had more potent ADCC activity than the monospecific formats, which could result in stronger tumor elimination.

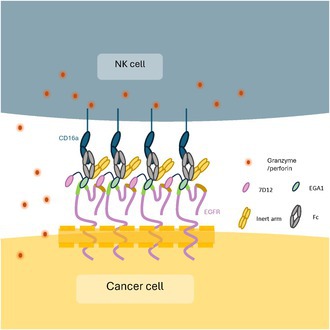

**Abbreviations**
ADCC: antibody‐dependent cell‐mediated cytotoxicity
BLI: biolayer interferometry
EC_50_
: half effective concentration
EDTA: ethylene diamine tetra acetic acid
EGFR: epidermal growth factor receptor
Em: emission
Ex: excitation
Fab: antigen‐binding fragment
FBS: fetal bovine serum
Fc: fragment crystallizable
FcγR: Fc gamma receptors
gMFI: geometric mean fluorescence intensity
HPLC: high performance liquid chromatography
IgG1: immunoglobulin G1
NK cell: natural killing cell
PBMCs: peripheral blood mononuclear cells
PBS: phosphate buffered solution
SEC: size exclusive chromatography
VHH: variable domains of the heavy chain of a heavy chain‐only antibody

The antibody‐dependent cell‐mediated cytotoxicity or antibody‐dependent cellular cytotoxicity (ADCC) is an immune defense mechanism in which immune effector cells are directed to kill target cells [1, 2, 3]. The recruitment of immune cells can include natural killer cells, macrophages, neutrophils, and eosinophils to boost a patient's defense against pathogens, dysfunctional cells, and cancer cells. ADCC is initiated after several sequential steps: antibodies binding to specific antigens on the surface of target cells for presentation of the antibody fragment crystallizable (Fc) domain; clustering of the antibody Fc domains on the target cell surface to coordinate binding by Fc gamma receptors (FcγR) on immune cells and/or complement factors; activation of immune cells that result in the release of cytotoxic molecules to destroy the target cells; and/or formation of complement protein clustering that results in target cell death [4, 5]. Thus, ADCC can play a critical role in the mechanism of action for multiple therapeutic antibodies that include cetuximab [6], daratumumab [7, 8], trastuzumab [9], and rituximab [10, 11].

The strength of ADCC depends on multiple factors, including the expression level of the target antigen on the cell surface, the selection of epitopes that present the Fc domain of the antibody, the binding of the antibody to the target antigen that enables efficient clustering on the cell surface, and the quantity of active effector cells in the diseased area [5, 12, 13, 14, 15, 16]. In addition, antibody Fc engineering can modulate ADCC activity by controlling antibody glycosylation (e.g., defucosylation of the antibody Fc domain to enhance FcγRIIIa binding) and modification of Fc affinity for effector cell receptors [3, 4, 5, 12, 17].

Here, we describe the coupling between target antigen engagement and Fc presentation on ADCC activity. Specifically, we investigated antibody binding to epidermal growth factor receptor (EGFR; UniProt P00533), a validated extracellular oncogenic target, and its functional impact on ADCC activity. EGFR is a 170 kDa transmembrane glycoprotein that plays a key role in cell growth [18, 19]. Overexpression or mutation of EGFR is closely linked to the development, growth, metastasis, and prognosis of various solid tumors, such as breast, ovarian, prostate, and non‐small‐cell lung cancer (NSCLC) [19, 20]. Consequently, anti‐EGFR antibodies, such as cetuximab and panitumumab, demonstrated efficacy in inhibiting tumor growth and have become cancer therapies for patients [21, 22, 23]. However, the therapeutic efficacy of these single‐epitope monoclonal antibodies (mAbs) is often limited by acquired resistance mechanisms and dose‐limiting toxicities, hindering sustained cancer remission [24, 25]. Alternatively, a tandem VHH‐based antibody approach can engage EGFR at distinctive epitopes simultaneously [26, 27]. As published before, fusing two anti‐EGFR domains into a single therapeutic molecule can enhance binding affinity and improve the inhibition of cancer cell signaling pathways [28, 29].

This study explored how dual VHH domain binding to EGFR influenced ADCC function. The two anti‐EGFR VHHs, 7D12, and EGA, which target distinct EGFR epitopes, were engineered into monovalent, bivalent, and biparatopic formats (fused to Fc). We described how a bispecific antibody with tandem anti‐EGFR VHHs 7D12 and EGA1 could have superior efficacy to the anti‐EGFR (i.e., cetuximab) monoclonal antibodies (mAbs) but with lower production costs that could be inherent in other asymmetric bispecific antibodies [27, 30, 31, 32, 33]. Additionally, such constructs that fuse the targeting VHH domain with a human IgG1 Fc can also significantly increase serum half‐life when compared to the VHH domain alone [28]. We demonstrated how the antigen‐binding site, valency, and avidity critically affected cell binding and ADCC efficacy. Notably, the ADCC activity of the tandem fusion of 7D12 and EGA1 was compared across different cell lines with varying cell surface EGFR densities.

## Materials and methods

### Cell lines and culture media

The cell lines and cell culture media used in this study were summarized in Table 1. The RKO, HCC827, and NCI‐H1975 cell lines were purchased from the China National Collection of Authenticated Cell Cultures. The SNU‐5, BxPC‐3, and HT‐29 cell lines were purchased from ATCC. The NCI‐N87, Capan‐2, Hs 578T, MDA‐MB‐231, and NCI‐H196 cell lines were purchased from Cobioer Bioscience Company, Ltd. More information on the cell lines with cellosaurus identification is described in Table 1. The cell lines were authenticated by the respective vendors. All experiments were performed with mycoplasma‐free cells. The adherent cells were passaged upon trypsin–EDTA digestion, while the suspension cell line SNU‐5 was passaged upon dilution.

**Table 1:** The information of cell lines. The catalog number link is embedded for each cell line used.

Cell name	Abstract	Source	Catalog number / Cellosaurus accession name^a^	Cell culture conditions	Cell culture media
RKO	Human colorectal adenocarcinoma cells	National Collection of Authenticated Cell Cultures	TCHu116/CVCL_0504	Adherent	EMEM+10% FBS
SNU‐5	Derived from ascites in a 33‐year‐old female with poorly differentiated gastric cancer	ATCC (American Type Culture Collection)	CRL‐5973/CVCL_0078	Suspension	IMDM +20% FBS
HCC827	Non‐small cell lung cancer cells from a 39‐year‐old female	National Collection of Authenticated Cell Cultures	SCSP‐538/CVCL_2063	Adherent	1640+Glutamax+10%FBS
NCI‐H1975	Human non‐small cell lung cancer cells	National Collection of Authenticated Cell Cultures	SCSP‐597/CVCL_1511	Adherent	1640+Glutamax+10%FBS
BxPC‐3	Epithelial cells from a 61‐year‐old woman with pancreatic cancer	ATCC (American Type Culture Collection)	CRL‐1687/CVCL_0186	Adherent	RPMI‐1640 Medium+10% FBS
HT‐29	44‐year‐old female with adenocarcinoma; Colorectal epithelial cells	ATCC (American Type Culture Collection)	HTB‐38/CVCL_0320	Adherent	McCoy's 5a Medium Modified+10% FBS
NCI‐N87	Human gastric carcinoma cell	Cobioer	CBP60491/CVCL_1603	Adherent	RPMI1640 + 10%FBS
Capan‐2	56‐year‐old male with pancreatic cancer cells, tissue of origin: pancreas	Cobioer	CBP60834/CVCL_0026	Adherent	IMDM+20%FBS
Hs 578 T	74‐year‐old woman with breast cancer	Cobioer	CBP60377/CVCL_0332	Adherent	DMEM+10%FBS + 0.01 mg/mL insulin
MDA‐MB‐231	Breast cancer epithelial cells from a 51‐year‐old woman	Cobioer	CBP60382/CVCL_0062	Adherent	DMEM+10% FBS
NCI‐H196	Human small cell lung cancer cells	Cobioer	CBP60120/CVCL_1509	Adherent	RPMI‐1640+10%FBS

^a^ Bairoch A. The Cellosaurus, a cell line knowledge resource. J. Biomol. Tech. 29:25–38(2018) DOI: 10.7171/jbt.18‐2902‐002; PMID: 29805321.

### Antibodies

E‐1 was the anti‐EGFR nanobody 7D12 fused to an IgG1 Fc portion. E‐2 was the anti‐EGFR nanobody EGA1 fused to an IgG1 Fc portion. E‐12 was the fusion of 7D12 in tandem with EGA1 using a (GGGGS)_2_ linker and then fused to a human IgG1 Fc (UniProt Accession ID: P0DOX5). These antibodies were expressed transiently in Expi293F cells (ThermoFisher #A14527CN). The constructs were purified using a MabSelect Sure Column with elution using 0.1 m Glycine‐HCl (pH 3.2) and neutralization with 1 m Tris–HCl (pH 9.0). After being polished by Superdex 200 column chromatography, monomeric and monodisperse molecules with purity above 95% via size exclusion chromatography (SEC) were obtained. The E‐1/N bispecific antibody (BsAb) was generated by combining E‐1 and negative control inert arm IgG1 antibody by the controlled Fab‐arm exchange process [34, 35, 36, 37]. The levels of bispecific antibody content were confirmed using the Bio Mix SEC. The E2/N and E12/N control BsAbs were generated in the same way as the E1/N BsAb.

### Cell binding assay

The binding of antibodies to cells was measured by flow cytometry. Briefly, the adherent cells were harvested using a cell dissociation solution (ThermoFisher #13151014; Waltham, MA, USA) and resuspended in PBS containing 2% (w/v) FBS. The resuspended cells were incubated with the test antibodies for 1 h on ice. After washing, a secondary antibody against human Fc conjugated with AF647(Ex: 638 nm/Em: 647 nm; Jackson ImmunoResearch, #109‐605‐190; West Grove, PA, USA) was diluted to 1:500 in PBS solution with 2% (w/v) FBS and used to resuspend the cells. After the cells were incubated on ice for 30 min in the dark, the cells were washed and analyzed by flow cytometry (Beckman CytoFLEX, Brea, CA, USA).

The binding of CD16a to cells was measured by flow cytometry. Briefly, the adherent cells were harvested using a cell dissociation solution (ThermoFisher # 13151014) and resuspended in PBS containing 2% (w/v) FBS. The resuspended cells were incubated with the test antibodies for 1 h on ice. After washing, the human CD16a‐biotinylated conjugate (Acro biosystems # CDA‐H82E9, Newark, DE, USA) was added and incubated on ice for 1 h. After washing, the detection antibody SA‐AF488 (Ex: 495 nm/Em: 519 nm, ThermoFisher #S32354) was added to the cell suspension and incubated on ice for 0.5 h. After incubation, the cells were washed and analyzed by flow cytometry (Beckman CytoFLEX). The cell binding data were analyzed with the cytexpert 2.4 software (Beckman Coulter). The gating strategy was conducted by setting the cells with right forward scatter (FSC) gated as P1‐ > single cells with right FSC‐A and the side scatter (SSC) based on FSC‐H values gated as P2‐ > the geometric mean fluorescence intensity (gMFI) of P2 cells. The values were analyzed by the cytexpert 2.4 software.

### ADCC reporter assay

The antibody ADCC activity was measured using a reporter assay based on the Jurkat‐FcγRIIIa V158 effector cells purchased from Vazyme (Nanjing Vazyme Biotech Company Limited, Nanjing, China). The experiments were conducted according to protocols provided by Vazyme. The target cells were seeded in 96‐well plates at a density of 12 500 cells per well, and ADCC reporter cells were added to the plates at 75 000 cells per well. Antibodies were added to the wells at various concentrations obtained by threefold serial dilutions and incubated at 37 °C for 6 h. The substrate of the reporter assay (Vazyme) was added to the plate, and the luminescence was measured using a plate reader (Tecan Spark).

### Binding mode prediction

The structure for 7D12‐EGA1 × inert arm (E12/N) bispecific antibody was modeled using a crystal structure of an intact human IgG1 antibody (PDB ID: 1HZH, UniProt Accession ID: P0DOX5), as well as a predicted 7D12‐EGA1‐Fc fusion heavy chain structure (https://yanglab.qd.sdu.edu.cn/trRosetta/). The structure for tandem VHHs binding to EGFR was predicted and modeled using crystal structures of VHH domains EGA1 (PDB ID: 4KRO) and 7D12 (PDB ID: 4KRL) in complex with the extracellular region of EGFR, as well as a predicted full‐length EGFR structure (AF‐PF00533‐F1, https://alphafold.ebi.ac.uk/entry/P00533). All structural figures were prepared in chimerax (https://www.rbvi.ucsf.edu/chimerax).

### Quantification and statistical analysis

Nonlinear analyses and the plots of the log‐transformed data regression Slope (log[agonist]; Hill Slope; 4 parameters) were performed using the GraphPad Prism software version 9.3.1 (GraphPad Software, San Diego, California, USA). The nonlinear regression was performed with least squares regression and using a no weighting method. The half‐maximal effective concentration (EC_50_) value was determined as the concentration of agonist that gives a half‐maximal response. Both EC_50_ and 95% CI (confidence interval) values were calculated by the graphpad prism software.

## Results

### Bivalent 7D12 demonstrated optimal ADCC activity among all formats tested

**Fig. 1:**
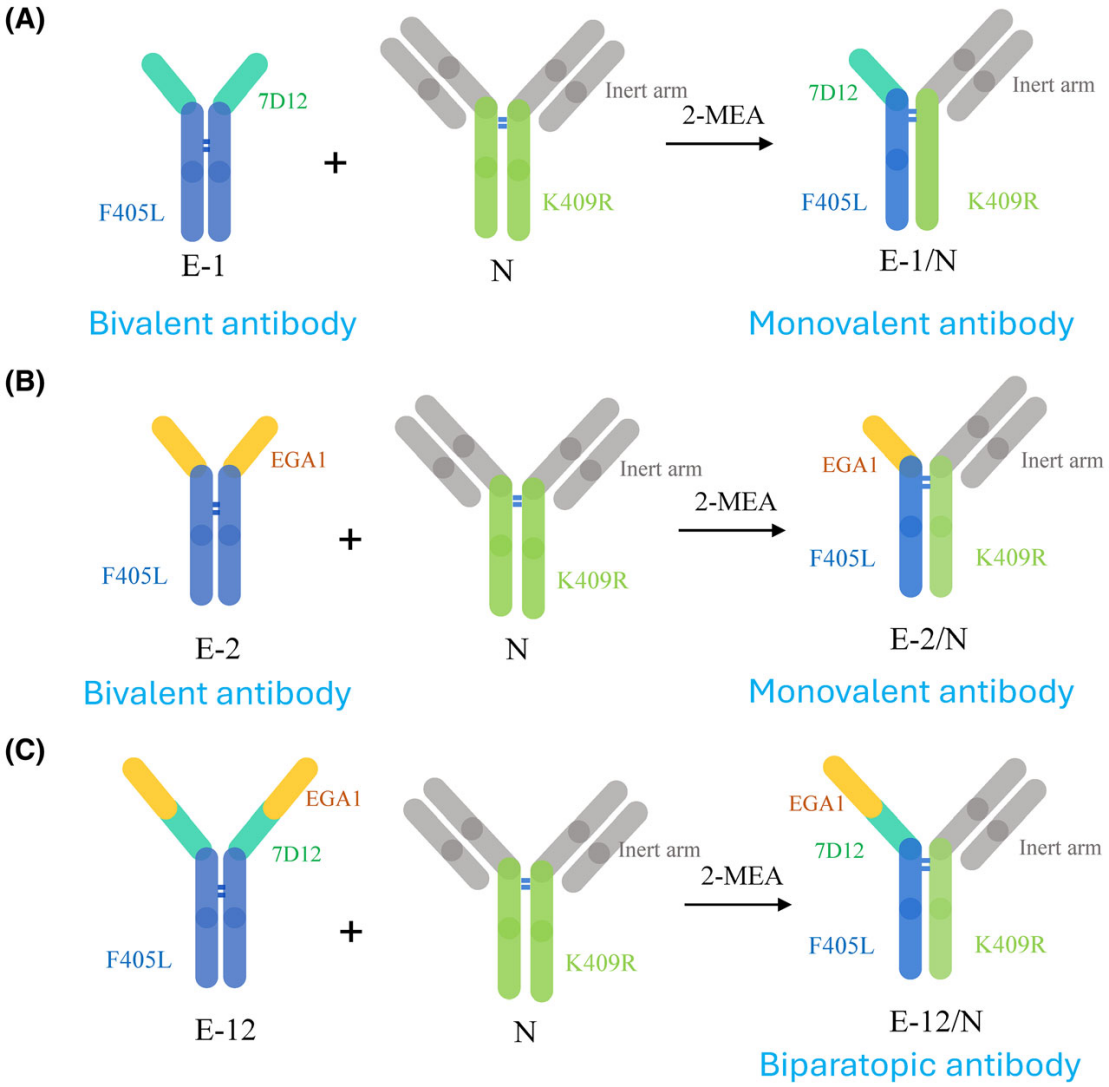
Schematic structures of antibodies. EGFR binding antibodies can replace one of its arms with an inert arm (Fab arms colored grey) through the controlled fab arm exchange (cFAE) method [35], thus producing the respective bispecific antibodies. (A) Monovalent antibody E‐1/N was derived from bivalent antibody E‐1, which was the anti‐EGFR nanobody 7D12 fused to an IgG1 Fc region, and the null antibody using the cFAE reaction. (B) Monovalent antibody E‐2/N was derived from bivalent antibody E‐2, which was the anti‐EGFR nanobody EGA1 fused to an IgG1 Fc region, and the null antibody using the cFAE reaction. (C) Monovalent biparatopic antibody E‐12/N was derived from E‐12, which was the fusion of 7D12 in tandem with EGA1 using a (GGGGS)_2_ linker and then fused to a human IgG1 Fc and the null antibody using the cFAE reaction.

The range of architectural variations of EGFR antibodies with different valencies and orientations is presented in Fig. 1. The anti‐EGFR nanobodies 7D12 and EGA1 were fused to a human IgG1 backbone and named E‐1 and E‐2, respectively. The N referred to the inert mAb with the similar human IgG1 isotype antibody that was similar to the IgG1 framework used for the parental E‐1 and E‐2 antibodies. The E‐1/N and E‐2/N were bispecific antibodies generated by using the controlled Fab‐arm exchange [34, 35, 36, 37] using E‐1 or E‐2 and N as parental mAbs (Fig. 1A and B). In order to compare the difference between monovalent and bivalent antibodies, the E‐1 and E‐2 were used as the bivalent mAb controls, and the E‐1/N and E‐2/N (the inert N arm did not bind to the target cell surface) BsAbs were used as monovalent antibodies.

All the antibodies had good transient expression titers (approximately 300 μg·mL^−1^), good SEC purity (> 90%), (Table S1 and Fig. S1), and good stability against aggregation for more than 3 months. Flow cytometry was employed to detect cell binding, while a reporter gene assay was used to measure the ADCC effect of these antibodies. Eleven cell lines spanning non‐small‐cell lung cancer, gastric cancer, colon cancer, breast cancer, and pancreatic cancer were tested. These cell lines have diverse EGFR density levels on the respective cancer cell lines (Table S2).

**Fig. 2:**
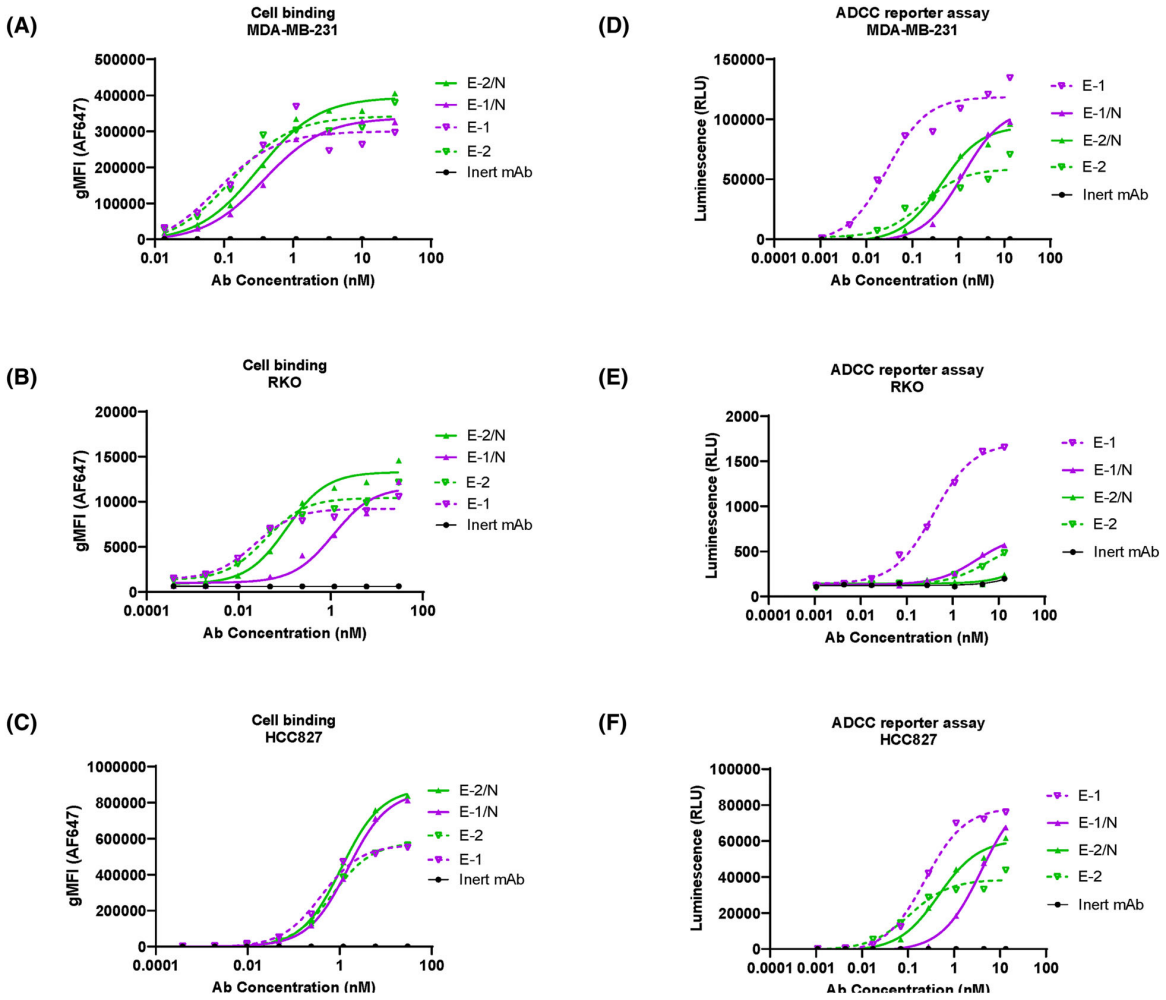
Cell binding and ADCC reporter assays of monovalent and bivalent antibodies. Bivalent antibodies E‐1 and E‐2 were compared with monovalent antibodies E‐1/N and E‐2/N in cell binding (A–C) and ADCC reporter assay (D–F). The inert antibody was used as a negative control. Triple negative breast cancer cell line MDA‐MB‐231 (A and D), colon cancer cell line RKO (B and E) and lung cancer cell line HCC827 (C and F) were tested. For cell binding (A–C), the *x*‐axes represent antibody concentrations, and the *y*‐axes show geometric mean fluorescence intensity (gMFI) of antibodies bound to cell surfaces. The binding experiments were done in triplicate. For ADCC reporter assays (D–F), the *x*‐axes indicate antibody concentrations, and the *y*‐axes display luminescence values. The ADCC reporter assays were done in triplicate.

The binding and ADCC reporter results of the representative cell lines, MDA‐MB‐231, RKO, and HCC827 with low, medium, and high levels of EGFR expression, were exhibited in Fig. 2. The results of the other cell lines were similar to what was shown in Fig. 2 and displayed fully in the Fig. S2. The complete list of EC_50_ and gMFI values is tabulated in Table S3.

Compared to E‐1/N, the bivalent antibody (E‐1) exhibited stronger cell binding potency (a smaller cell binding EC_50_ in most cell lines) (Fig. 2A–C), suggesting the presence of avidity binding [13]. However, the difference in EC_50_ was not significant in some cell lines, which could be influenced by other cell surface factors such as glycosylation. In this study, we analyzed the relationship between EGFR levels and cell binding EC_50_ across 11 cell lines and found no significant correlation (data not shown). Interestingly, the monovalent antibody (E‐1/N) exhibited higher cell binding efficacy (maximal gMFI) (Fig. 2A–C). The E‐1/N monovalent binding had a higher density of cell surface binding under limited surface antigen conditions compared to the bivalent (E‐1) format. Consequently, there could be greater Fc region exposure on the cell surface. Notably, the increased Fc presentation of E‐1/N did not translate into a stronger ADCC activity in ADCC reporter assays. On the contrary, the bivalent antibody E‐1 generated significantly stronger ADCC signals than its monovalent counterpart E‐1/N (Fig. 2D–F). Thus, the occurrence of ADCC was influenced not only by the quantity of antibody Fc bound to the cell surface but also likely by other factors, such as the arrangement of antibodies on the cell surface, and the orientation of the Fc region. Similar cell binding patterns were observed between E‐2 and E‐2/N (Fig. 2A–C). However, E‐2/N consistently generated stronger ADCC signals than E‐2 across most cell lines, likely attributable to its enhanced antibody presentation (Fig. 2D–F).

The 7D12 and EGA1 nanobodies target distinct EGFR epitopes [30]. We consequently compared their cell binding and ADCC activities in both bivalent and monovalent formats. While the E‐2 and E‐2/N demonstrated marginally stronger cell binding than E‐1 and E‐1/N, respectively, the ADCC activity revealed an inverse relationship: E‐1 showed significantly greater ADCC than E‐2, and E‐1/N produced slightly higher maximal signal than E‐2/N. These results indicated that the ADCC efficacy depended on epitope specificity, not merely on binding avidity.

### The EGA1‐7D12 biparatopic EGFR antibody exhibited superior cell binding and ADCC activity compared to the monovalent single‐epitope antibodies

**Fig. 3:**
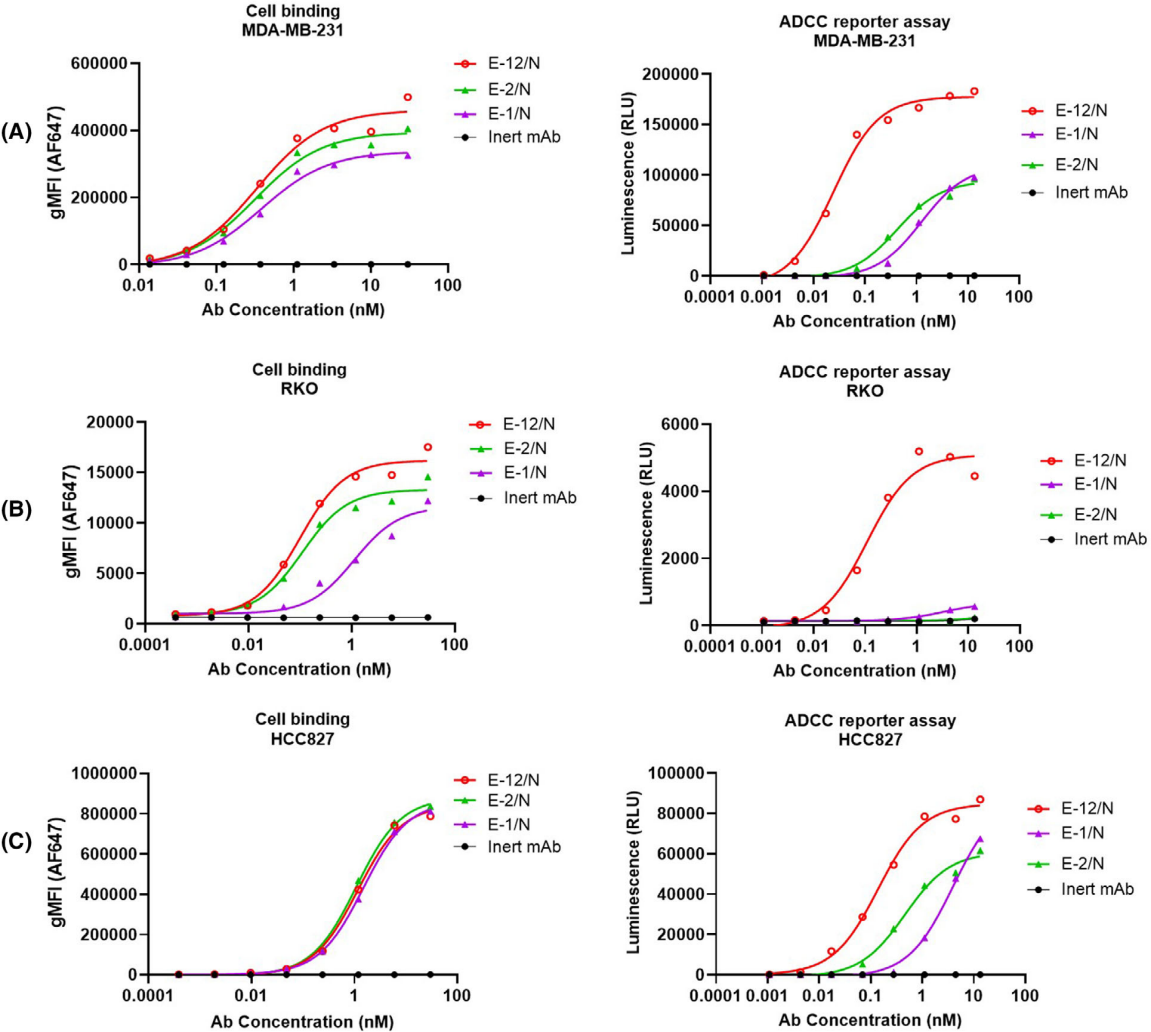
Cell binding and ADCC reporter of monovalent single epitope and biparatopic antibodies. Biparatopic E‐12/N was compared with monovalent single paratopic antibodies E‐1/N and E‐2/N in cell binding (A–C) and ADCC reporter assay (D–F). The inert antibody was used as a negative control. Triple‐negative breast cancer cell line MDA‐MB‐231 (A and D), colon cancer cell line RKO (B and E) and lung cancer cell line HCC827 (C and F) were tested. For cell binding (A–C), the *x*‐axes represent antibody concentrations, and the *y*‐axes show geometric mean fluorescence intensity (gMFI) of antibodies bound to cell surfaces. The binding experiments were done in triplicate. For ADCC reporter assays (D–F), the *x*‐axes indicate antibody concentrations, and the *y*‐axes display luminescence values. The ADCC reporter assays were done in triplicate.

Our results demonstrated that 7D12 mediated stronger ADCC activity than EGA1, likely due to epitope specificity rather than binding affinity. Based on this finding, we next explored whether a dual‐epitope format could further improve ADCC function. To confirm this hypothesis, we generated the biparatopic EGFR antibody E‐12/N through controlled Fab‐arm exchange [34, 35, 36, 37], using the parental antibody E‐12 and an inert control antibody (Fig. 1C). The resulting construct, containing tandem 7D12 and EGA1 VHH domains and an inert arm, demonstrated favorable biophysical properties, including high titer in transient expression, > 90% purity by SEC analysis, and excellent stability. (Table S1 and Fig. S1). All antibodies demonstrated similar CD16a (FcγRIIIA) binding affinity, attributable to their shared Fc framework (Table S4). Next, we evaluated both cell binding capacity using flow cytometry and ADCC activity through a reporter assay. Representative results of MDA‐MB‐231, RKO, and HCC827 cell lines with low, medium, and high levels of EGFR expression are exhibited in Fig. 3. The results of the other cell lines with comparable profiles are shown in Fig. S3. Overall, the results revealed that while E‐12/N showed only modest improvements in cell binding (gMFI) over its monovalent single‐epitope counterparts (E‐1/N and E‐2/N), it achieved substantially enhanced ADCC potency and efficacy, with significantly lower EC_50_ values and higher maximal signal levels (Fig. 3 and Table S3). In summary, while the E‐1/N and E‐2/N BsAbs had relatively weak ADCC activity, E‐12/N, which had both 7D12 and EGA1 binding arms, mediated higher ADCC responses. Thus, there could be other mechanisms besides antibody binding levels that could drive ADCC activity.

### The EGA1‐7D12 biparatopic EGFR antibody exhibited superior cell binding and ADCC activity compared to the bivalent single‐epitope antibodies

**Fig. 4:**
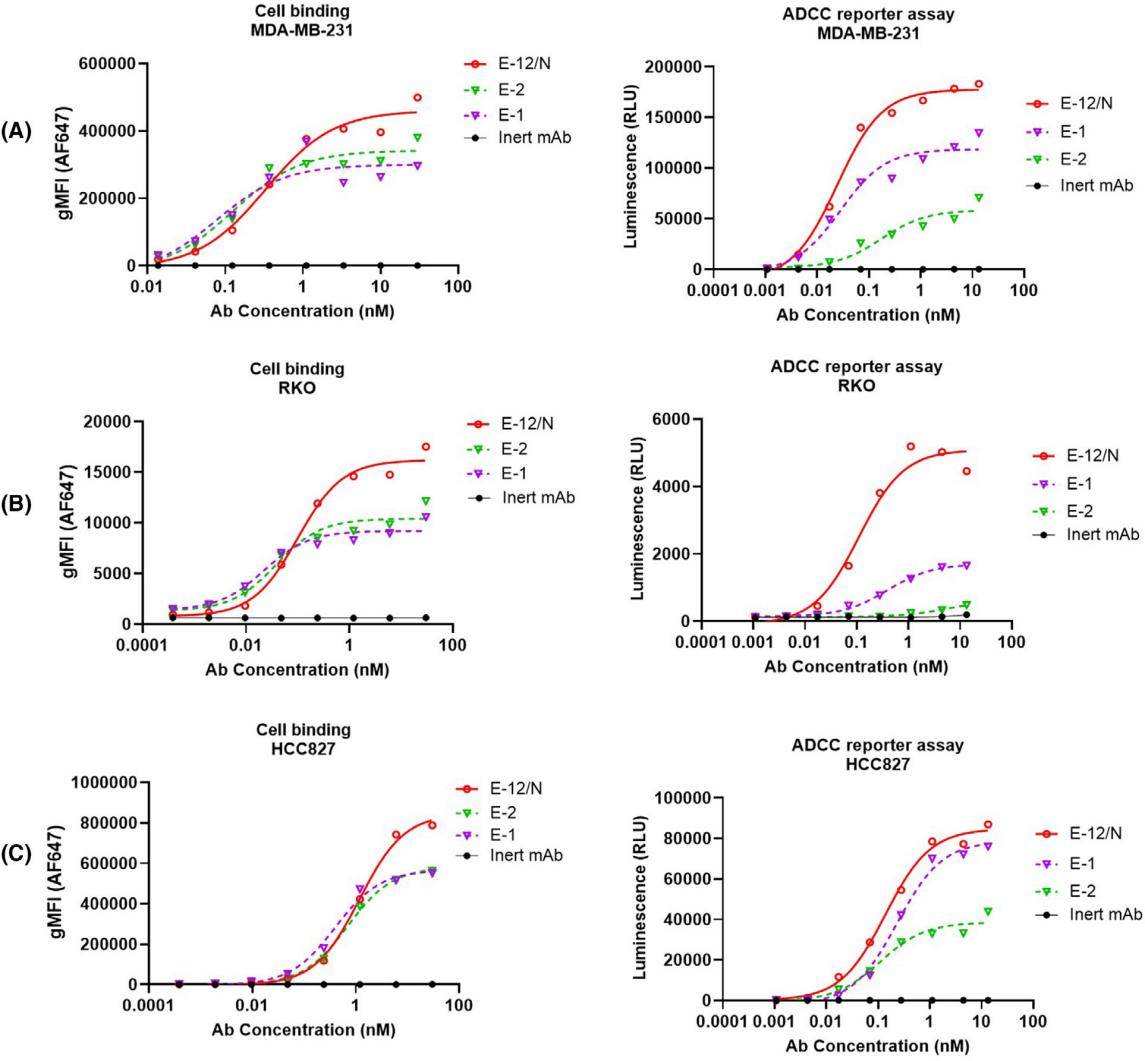
Cell binding and ADCC reporter of biparatopic and bivalent antibodies. Biparatopic antibody E‐12/N was compared with bivalent antibodies E‐1 and E‐2 in cell binding (A–C) and ADCC reporter assay (D–F). The inert antibody was used as a negative control. Triple‐negative breast cancer cell line MDA‐MB‐231 (A and D), colon cancer cell line RKO (B and E) and lung cancer cell line HCC827 (C and F) were tested. For cell binding (A–C), the *x*‐axes represent antibody concentrations, and the *y*‐axes show geometric mean fluorescence intensity (gMFI) of antibodies bound to cell surfaces. The binding experiments were done in triplicate. For ADCC reporter assays (D–F), the *x*‐axes indicate antibody concentrations, and the *y*‐axes display luminescence values. The ADCC reporter assays were done in triplicate.

While the combination of distinct anti‐EGFR nanobodies synergistically enhanced ADCC activity, we then examined a comparison between the biparatopic construct and E‐1 (the top‐performing) bivalent monospecific antibody across all valency formats (Fig. 1). The biparatopic antibody E‐12/N and analogous bivalent antibodies E‐1 and E‐2 were compared for cell binding via flow cytometry (Fig. 4A–C) and ADCC via a reporter gene assay (Fig. 4D–F). The results showed that the biparatopic antibody E‐12/N demonstrated superior cell binding (higher gMFI) while maintaining comparable EC_50_ values relative to bivalent antibodies E‐1 and E‐2 (Fig. 4A–C). However, the E‐12/N BsAb exhibited significantly enhanced ADCC activity in reporter assays compared to E‐1 (Figs 4, S4 and Table S3). These results suggested that E‐12/N engaged EGFR through a distinct binding mode that extended beyond simple avidity effects from bivalent binding. This unique interaction mechanism likely improved ADCC potency.

### The biparatopic EGFR antibody exhibited superior CD16a binding on cell surface

**Fig. 5:**
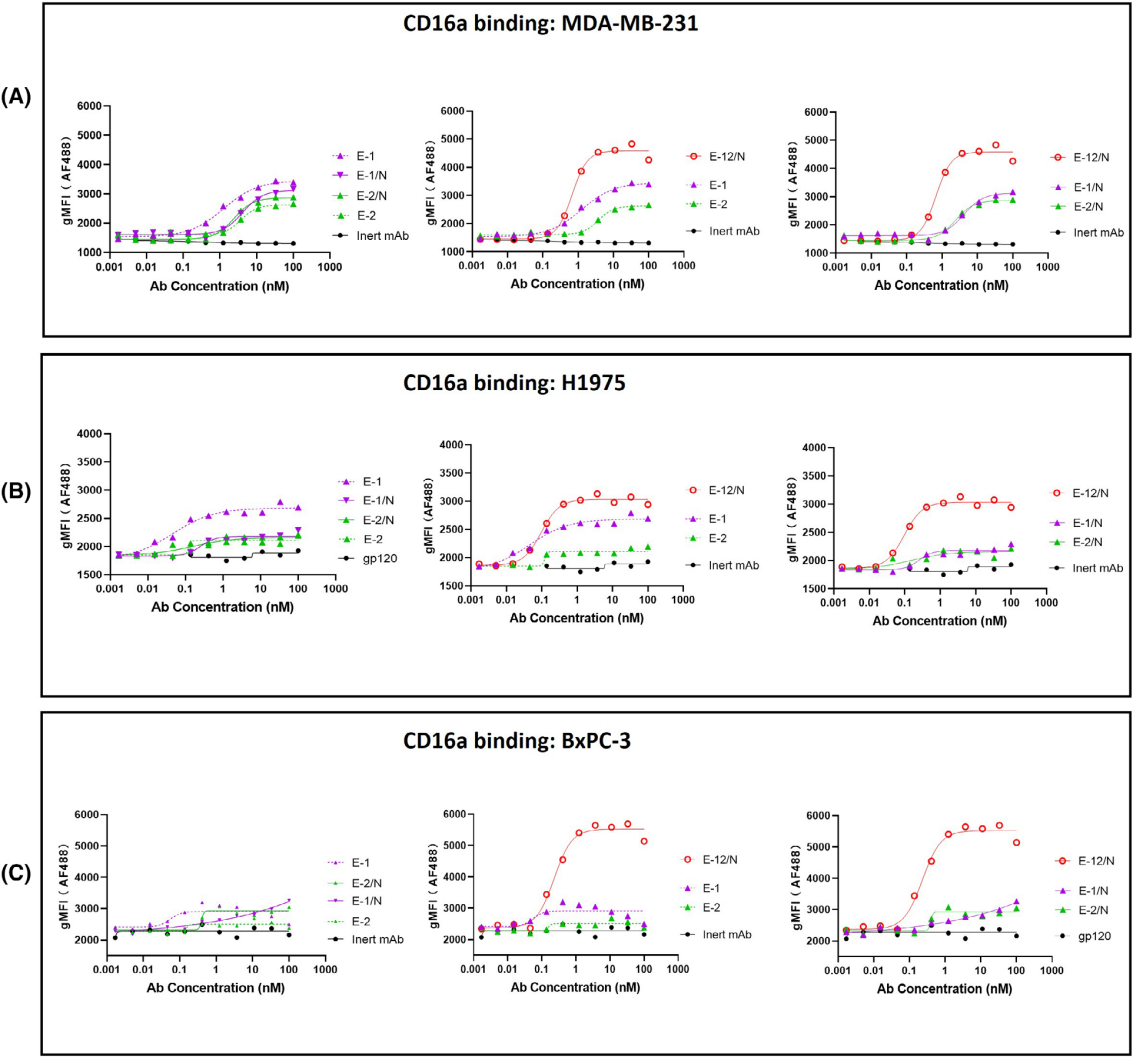
CD16a binding capacity detection. Three EGFR expression cell lines were used to test CD16a binding capacity of antibodies on the cell membrane. MDA‐MB‐231 served as the breast cancer sample (A–C), H1975 as the lung cancer sample (D–F), and BxPC‐3 as the pancreatic cancer sample (G–I). The *x*‐axes represent antibody concentrations, and the *y*‐axes show geometric mean fluorescence intensity (gMFI) of CD16a bound to the cell surface. The binding experiments were done in triplicate.

To better understand the mechanism of the tandem VHH construct enhanced ADCC activity, we probed the binding activity of the antibodies bound to the cells to CD16a. In the experiment, antibodies were first allowed to bind to the cells, followed by the addition of free biotin‐labeled CD16a, which then bound with the antibodies on the cell surface. Finally, fluorescently labeled streptavidin (SA) was added to reflect the quantity of bound CD16a through fluorescence signals. Three cell lines, MDA‐MB‐231 (Fig. 5A–C), H1975 (Fig. 5D–F), and BxPC‐3 (Fig. 5G–I), were probed in parallel. While E‐1 and E‐2 had comparable cell binding levels, the bivalent antibody E‐1 exhibited stronger CD16a binding than E‐2 which could explain why E‐1 mediated a much stronger ADCC response. The biparatopic antibody E‐12/N demonstrated even greater CD16a engagement compared to the bivalent E‐1 thereby corroborating the respective ADCC activities. In summary, E‐12/N demonstrated superior ADCC activity, likely attributable to the enhanced CD16a engagement following cell surface binding compared to those of the monospecific antibody formats. This could be due to a unique interaction of the biparatopic antibody with EGFR on the cell surfaces.

### The two EGFR binding arms of E‐12/N exhibited a preference for binding to distinct EGFR molecules rather than the same EGFR molecule

**Fig. 6:**
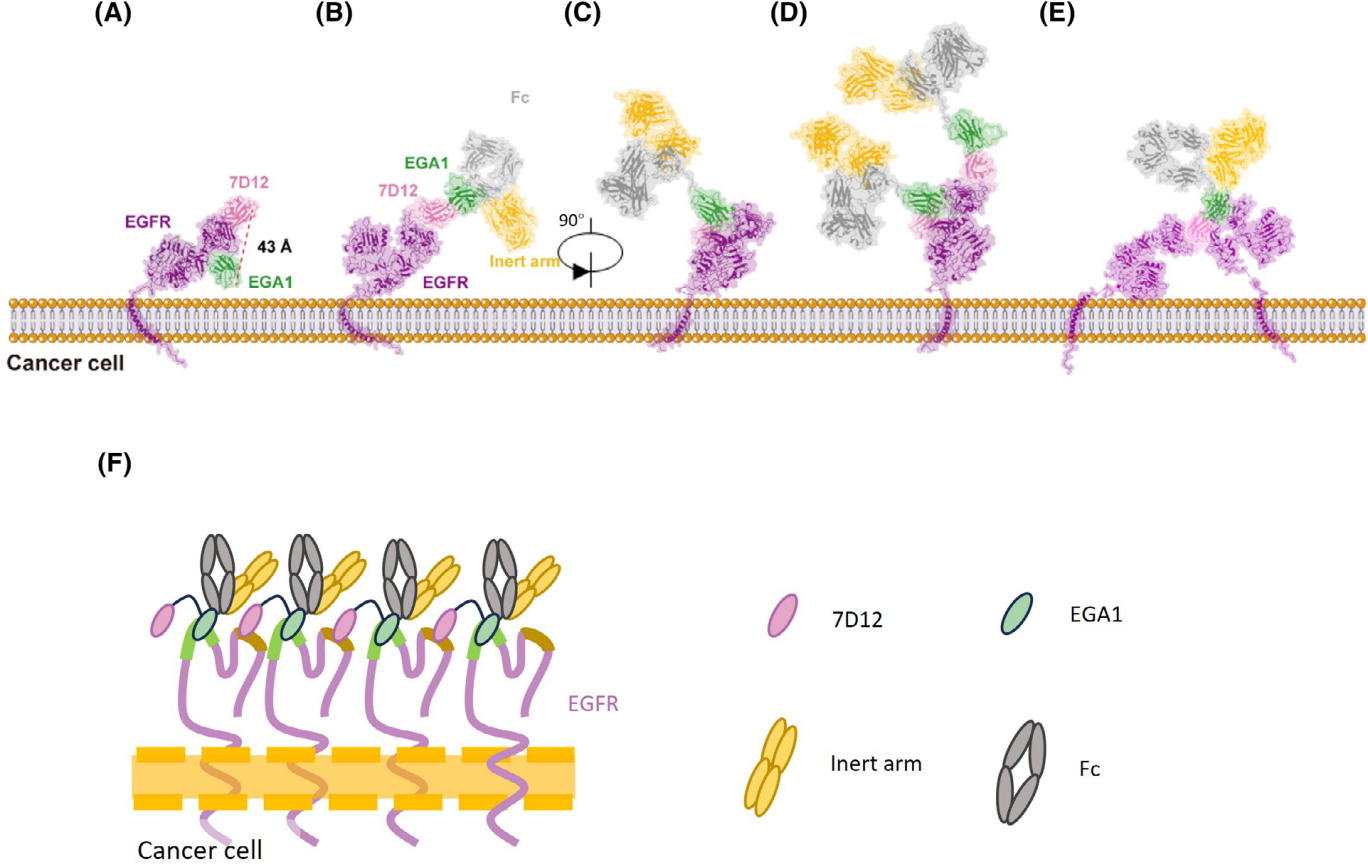
Structural basis for tandem VHHs binding to EGFR. (A) The predicted binding mode of 7D12 and EGA1 VHHs to membrane‐bound EGFR simultaneously. A red dashed line indicates the distance (43 Å) between the 7D12 VHH C terminus and the EGA1 VHH N terminus. (B and C) The predicted binding mode of 7D12‐EGA1 × inert arm bispecific antibodies (E‐12/N) binding to membrane‐bound EGFR at a 1 : 1 molar ratio. (D) The predicted binding mode at a 2 : 1 molar ratio. (E) The predicted binding mode at a 1 : 2 molar ratio. (F) The predicted binding mode of multiple E12/N antibodies binding to EGFR and forming multiple complexes at the cell membrane. EGFR, 7D12, and EGA1 VHHs are colored purple, pink, and green, respectively. The Fc and inert arm are colored gray and orange, respectively. All structures are shown in cartoon and surface representations.

The structure for tandem VHHs binding to EGFR was predicted and modeled using crystal structures of VHH domains EGA1 (PDB ID: 4KRO) and 7D12 (PDB ID: 4KRL) in complex with the extracellular region of EGFR, as well as a predicted full‐length EGFR structure (AF‐PF00533‐F1, https://alphafold.ebi.ac.uk/entry/P00533). Based on the predicted crystal structure, the distance between the C terminus of 7D12 VHH and the N terminus of EGA1 VHH when binding to one EGFR was 43 Å (Fig. 6A). The E‐12/N BsAb had the 7D12 and EGA1 domains connected via a GGGGSGGGGS linker. The GGGGS amino acid sequence is known to form a flexible linker spanning 19 Å between two proteins [38, 39]. Since the distance between 7D12 and EGA1 VHHs (~ 38 Å) was shorter than 43 Å, the tandem VHHs on one arm were not likely to bind to a single EGFR molecule.

The structure for E‐12/N was modeled using a crystal structure of an intact human IgG1 antibody (PDB ID: 1HZH), as well as a predicted 7D12‐EGA1‐Fc fusion heavy chain structure (https://yanglab.qd.sdu.edu.cn/trRosetta/). A potential binding model for E‐12/N and EGFR could be a molar ratio of 1 : 1, 2 : 1, or 1 : 2 (Fig. 6B–E): the 7D12‐EGA1 × inert arm bispecific antibody binds to a membrane‐bound EGFR either through the 7D12 (Fig. 6B) or EGA1 (Fig. 6C) binding domain. Alternatively, two E‐12/N antibodies could bind to a membrane‐bound EGFR through the 7D12 and EGA1 binding domains, respectively (Fig. 6D). Alternatively, the 7D12 in E‐12/N could bind to one EGFR while EGA1 bound to another EGFR molecule, forming a 1 : 2 complex (Fig. 6E). Furthermore, we proposed a novel binding mode wherein E‐12/N antibodies bridged multiple EGFR molecules to form higher order complexes (Fig. 6F). In these complexes, Fc regions could be densely clustered on the cell membrane through EGFR oligomerization. We hypothesized that this spatial organization could facilitate multivalent CD16a engagement on NK cells, thereby amplifying ADCC responses.

## Discussion

ADCC is a critical mechanism through which therapeutic antibodies could better control disease progression [2]. Numerous studies describe how antibody ADCC function is enhanced by increasing antibody–antigen affinity, antibody glycosylation, and mutagenesis of Fc mutations to enhance affinity for Fc gamma receptors on effector cells [14, 15, 17, 40, 41, 42]. In addition, bispecific antibodies (BsAbs) can utilize Fab and Fc interactions to enhance affinity and avidity [43]. By fine‐tuning the binding properties of the two antigen‐recognition arms, BsAbs can achieve greater selectivity and cytotoxicity [44, 45]. Unlike conventional monoclonal antibodies (mAbs), which engage bivalently with target antigens, presenting a single Fc domain per bound cell, BsAbs with monovalent binding domains can recruit multiple Fc domains per target cell, thereby amplifying FcγR‐mediated effector functions [46]. Furthermore, the dual‐binding architecture of BsAbs enables diverse interaction modes with target cells, allowing for adjustable potency and immune activation [13]. Understanding the relationship between the binding mode of bispecific antibodies and ADCC activities provides the basis for improving the engineering of multispecific antibodies. In this study, we demonstrated how a bispecific antibody with dual anti‐EGFR binding arms enhanced ADCC activity.

We generated bivalent monospecific anti‐EGFR antibodies (E‐1 [7D12] and E‐2 [EGA1], targeting distinct epitopes), a biparatopic antibody (E‐12), and their corresponding monovalent variants using the controlled Fab‐arm exchange (Fig. 1) [34, 35, 36, 37]. The biolayer interferometry (BLI) results indicated that these antibodies had similar affinities for CD16a due to their identical antibody framework structures (Table S4). Based on these monovalent, bivalent, single‐epitope, and dual‐epitope EGFR antibodies, the effects of antigen binding epitope, antigen binding valency, and biparatopic binding avidity on cell binding and ADCC activity were revealed. In this study, we utilized an ADCC reporter assay instead of PBMC‐based methods to assess ADCC activity. This system provided a standardized platform to evaluate cell binding effects while circumventing donor‐specific variability inherent to PBMC cytotoxicity assays. The ADCC reporter assay not only mimicked the traditional PBMCs method but also provided a more precise and consistent performance [47, 48]. Through cell binding and ADCC reporting experiments, we evaluated antibody performance across 11 cancer cell lines (breast, colon, gastric, lung, and pancreatic) with varying EGFR expression levels, to elucidate the enhancement of ADCC activity (Tables 1 and S1).

Although E‐1 (7D12‐Fc) and E‐2 (EGA1‐Fc) showed comparable cell binding capabilities, E‐1 demonstrated significantly stronger ADCC activity. This functional difference likely stemmed from their distinct epitope specificities: 7D12 bound to a flat surface on EGFR domain III, while EGA1 targeted an epitope near the domain II/III junction [30]. Perhaps domain III binding enabled optimal Fc presentation and clustering, thereby enhancing CD16a engagement compared to domain II/III junction binding (Figs 2, S2 and 5). To our knowledge, this represented the first report delineating ADCC efficacy differences between these two anti‐EGFR VHHs (7D12 and EGA1), despite previous characterizations of their divergent biological properties [28, 29, 30, 49]. Interestingly, the bivalent binding boosted ADCC for the stronger 7D12 epitope, while the monovalent format worked better for the weaker EGA1 epitope (Figs 2 and S2). Such profiles suggested that monovalent antibody binding selection could improve Fc presentation when epitope engagement would be suboptimal. While EGA1 alone showed weak ADCC activity, its fusion with 7D12 (E12/N) synergistically enhanced ADCC. E12/N not only outperformed both E‐1/N and E‐2/N (Fig. 3), but also exceeded E1 and E2 in both potency and efficacy (Fig. 4). This enhancement could not be solely explained by improved cell binding (Figs 3 and 4), as the ADCC improvement significantly outpaced the modest increase in cell binding affinity, thereby suggesting additional mechanisms. Indeed, we found E‐12/N demonstrated stronger CD16a engagement when bound to cell‐surface EGFR (Fig. 5). These consistent results across all 11 tested cell lines indicated that the biparatopic design uniquely optimized both target engagement and effector function.

To elucidate antibody–receptor interactions, structural modeling of EGFR complexes with 7D12, EGA1, and E12/N was performed (Fig. 6). The model revealed that 7D12 and EGA1 in E12/N were unlikely to bind the same EGFR molecule due to steric constraints; rather preferential engagement could occur with two distinct EGFR molecules. This model hinted that E12/N induced a specific topological arrangement of membrane‐bound EGFR that could promote receptor clustering (Fig. 6F). This structural configuration could facilitate the clustering of antibody Fc, analogous to the C1q‐Fc interaction in complement activation [40, 41, 50, 51]. Our results were consistent with previous reports demonstrating that zanidatamab, a HER2‐targeting biparatopic antibody, enhanced CDC through receptor clustering [52].

This research offered insights into the development of more effective antibody therapies for targeting EGFR‐positive cancer cells. The findings indicated that the biparatopic antibody E12/N had the potential to interact with EGFR in a specific topological arrangement on the cell membrane, leading to the enhanced activation of NK cells and subsequent elimination of target cells. Understanding the mechanism of action of E12/N could pave the way for the development of novel antibody‐based treatments with improved efficacy in treating EGFR‐positive cancers. Additionally, engineering antibodies to have enhanced ADCC activity could advance targeted antibody therapies to be more effective treatments for cancer and other diseases.

## Conflict of interest

The authors are employees of Tavotek Biotherapeutics Inc. The authors declare no conflict of interest.

## Author contributions

YX designed the antibody constructions, conducted experiments and data analyses including CD16a cell binding, ADCC reporter assays, and prepared the original draft; HJ conducted the BLI binding and cell binding assays; HJ and LC participated in the expression, purification and SEC‐HPLC analysis of antibodies. FZ and YJ contributed to protein structure prediction and assisted with manuscript writing. MLC led the project conception, supervised the study, edited, and reviewed the manuscript. All authors approved the final version of the manuscript.

## Supporting information


**Table S1.** Amino acid sequences of the constructs.
**Table S2.** SEC‐HPLC purity of monoclonal and double antibodies.
**Table S3.** EGFR density on different cell lines.
**Table S4.** Cell binding and ADCC reporter assay values for the constructs.
**Table S5.** The affinity of antibody binding to human CD16a via the Octet (ForteBio).
**Fig. S1.** Monodispersity of antibodies.
**Fig. S2.** Cell binding and ADCC reporter assays of monovalent and bivalent antibodies.
**Fig. S3.** Cell binding and ADCC reporter of single paratopic and biparatopic antibodies.
**Fig. S4.** Cell binding and ADCC reporter of biparatopic and bivalent antibodies.

## Data Availability

The data in the manuscript are embedded in the Graphpad figure files in the Supporting Information. We reference sequences and structures: human IgG1 Fc (UniProt Accession ID: P0DOX5); human IgG1 antibody (PDB ID: 1HZH, UniProt Accession ID: P0DOX5); x‐ray crystal structures of VHH domains EGA1 (PDB ID: 4KRO) and 7D12 (PDB ID: 4KRL) in complex with the extracellular region of EGFR; full‐length EGFR structure (AF‐PF00533‐F1, https://alphafold.ebi.ac.uk/entry/P00533).
