# Supplementary material for: Engineering tandem VHHs to target different epitopes to enhance antibody‐dependent cell‐mediated cytotoxicity
Source: FEBS Open Bio. 2025 Nov 20;16(5):1030–43. doi: 10.1002/2211-5463.70166 (PMC13145349; doi:10.1002/2211-5463.70166)
Supplement: Supplementary file 1 — Table S1. Amino acid sequences of the constructs. Table S2. SEC‐HPLC purity of monoclonal and double antibodies. Table S3. EGFR density on different cell lines. Table S4. Cell binding and ADCC reporter assay values for the constructs. Table S5. The affinity of antibody binding to human CD16a via the Octet (ForteBio). Fig. S1. Monodispersity of antibodies. Fig. S2. Cell binding and ADCC reporter assays of monovalent and bivalent antibodies. Fig. S3. Cell binding and ADCC reporter of single paratopic and biparatopic antibodies. Fig. S4. Cell binding and ADCC reporter of biparatopic and bivalent antibodies. [file FEB4-16-1030-s001.docx]

**Supplemental information**

**Engineering Tandem VHHs Targeting Different Epitopes to Enhance Antibody-Dependent Cell-Mediated Cytotoxicity**

Yuqiang Xu^1^, Hao Jiang^1^, Limin Chen^1^, Fulai Zhou^1^, Ying Jin^1^, Mark L. Chiu^1, 2*^

^1^Research & Development Department, Tavotek Biotherapeutics, Suzhou 215000, China

^2^Research & Development, Tavotek Biotherapeutics, Spring House, Pennsylvania 19102, United States

^*^Corresponding author. Email: [mark.chiu@tavotek.com](mailto:mark.chiu@tavotek.com)

**Table S1: Amino acid sequences of the constructs**

| Construct | Format | Sequence (Heave chain) | Sequence (Light chain) |
| --- | --- | --- | --- |
| E-1 | 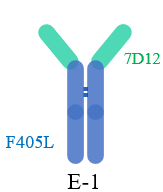 | QVKLEESGGGSVQTGGSLRLTCAASGRTSRSYGMGWFRQAPGKEREFVSGISWRGDSTGYADSVKGRFTISRDNAKNTVDLQMNSLKPEDTAIYYCAAAAGWAWYGTLYEYDYWGEGTQVTVSSGGGGSDKTHTCPPCPAPELLGGPSVFLFPPKPKDTLMISRTPEVTCVVVDVSHEDPEVKFNWYVDGVEVHNAKTKPREEQYNSTYRVVSVLTVLHQDWLNGKEYKCKVSNKALPAPIEKTISKAKGQPREPQVYTLPPSREEMTKNQVSLTCLVKGFYPSDIAVEWESNGQPENNYKTTPPVLDSDGSFLLYSKLTVDKSRWQQGNVFSCSVMHEALHNHYTQKSLSLSPGK | / |
| E-2 | 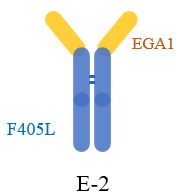 | QVQLQESGGGLVQPGGSLRLSCAASGRTFSSYAMGWFRQAPGKQREFVAAIRWSGGYTYYTDSVKGRFTISRDNAKTTVYLQMNSLKPEDTAVYYCAATYLSSDYSRYALPQRPLDYDYWGQGTQVTVSSGGGGSDKTHTCPPCPAPELLGGPSVFLFPPKPKDTLMISRTPEVTCVVVDVSHEDPEVKFNWYVDGVEVHNAKTKPREEQYNSTYRVVSVLTVLHQDWLNGKEYKCKVSNKALPAPIEKTISKAKGQPREPQVYTLPPSREEMTKNQVSLTCLVKGFYPSDIAVEWESNGQPENNYKTTPPVLDSDGSFLLYSKLTVDKSRWQQGNVFSCSVMHEALHNHYTQKSLSLSPGK | / |
| E-12 | 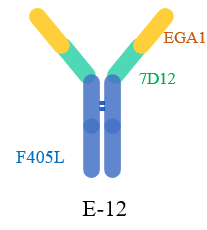 | QVQLQESGGGLVQPGGSLRLSCAASGRTFSSYAMGWFRQAPGKQREFVAAIRWSGGYTYYTDSVKGRFTISRDNAKTTVYLQMNSLKPEDTAVYYCAATYLSSDYSRYALPQRPLDYDYWGQGTQVTVSS**GGGGSGGGGS**QVKLEESGGGSVQTGGSLRLTCAASGRTSRSYGMGWFRQAPGKEREFVSGISWRGDSTGYADSVKGRFTISRDNAKNTVDLQMNSLKPEDTAIYYCAAAAGWAWYGTLYEYDYWGEGTQVTVSSGGGGSDKTHTCPPCPAPELLGGPSVFLFPPKPKDTLMISRTPEVTCVVVDVSHEDPEVKFNWYVDGVEVHNAKTKPREEQYNSTYRVVSVLTVLHQDWLNGKEYKCKVSNKALPAPIEKTISKAKGQPREPQVYTLPPSREEMTKNQVSLTCLVKGFYPSDIAVEWESNGQPENNYKTTPPVLDSDGSFLLYSKLTVDKSRWQQGNVFSCSVMHEALHNHYTQKSLSLSPGK | / |
| N | 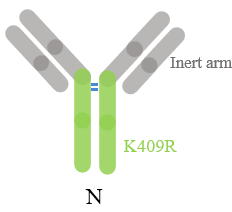 | QVQLVQSGAEVKKPGASVKVSCQASGYRFSNFVIHWVRQAPGQRFEWMGWINPYNGNKEFSAKFQDRVTFTADTSANTAYMELRSLRSADTAVYYCARVGPYSWDDSPQDNYYMDVWGKGTTVIVSSASTKGPSVFPLAPSSKSTSGGTAALGCLVKDYFPEPVTVSWNSGALTSGVHTFPAVLQSSGLYSLSSVVTVPSSSLGTQTYICNVNHKPSNTKVDKKVEPKSCDKTHTCPPCPAPEAAGGPSVFLFPPKPKDTLMISRTPEVTCVVVDVSHEDPEVKFNWYVDGVEVHNAKTKPREEQYNSTYRVVSVLTVLHQDWLNGKEYKCKVSNKALPAPIEKTISKAKGQPREPQVYTLPPSRDELTKNQVSLTCLVKGFYPSDIAVEWESNGQPENNYKTTPPVLDSDGSFFLYSRLTVDKSRWQQGNVFSCSVMHEALHNHYTQKSLSLSPGK | EIVLTQSPGTLSLSPGERATFSCRSSHSIRSRRVAWYQHKPGQAPRLVIHGVSNRASGISDRFSGSGSGTDFTLTITRVEPEDFALYYCQVYGASSYTFGQGTKLERKRTVAAPSVFIFPPSDEQLKSGTASVVCLLNNFYPREAKVQWKVDNALQSGNSQESVTEQDSKDSTYSLSSTLTLSKADYEKHKVYACEVTHQGLSSPVTKSFNRGEC |
| E-1/N | 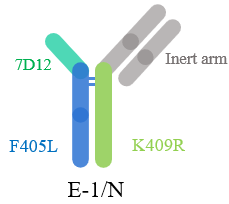 | 7D12 heavy chain construct  QVKLEESGGGSVQTGGSLRLTCAASGRTSRSYGMGWFRQAPGKEREFVSGISWRGDSTGYADSVKGRFTISRDNAKNTVDLQMNSLKPEDTAIYYCAAAAGWAWYGTLYEYDYWGEGTQVTVSSGGGGSDKTHTCPPCPAPELLGGPSVFLFPPKPKDTLMISRTPEVTCVVVDVSHEDPEVKFNWYVDGVEVHNAKTKPREEQYNSTYRVVSVLTVLHQDWLNGKEYKCKVSNKALPAPIEKTISKAKGQPREPQVYTLPPSREEMTKNQVSLTCLVKGFYPSDIAVEWESNGQPENNYKTTPPVLDSDGSFLLYSKLTVDKSRWQQGNVFSCSVMHEALHNHYTQKSLSLSPGK  Inert arm heavy chain construct  QVQLVQSGAEVKKPGASVKVSCQASGYRFSNFVIHWVRQAPGQRFEWMGWINPYNGNKEFSAKFQDRVTFTADTSANTAYMELRSLRSADTAVYYCARVGPYSWDDSPQDNYYMDVWGKGTTVIVSSASTKGPSVFPLAPSSKSTSGGTAALGCLVKDYFPEPVTVSWNSGALTSGVHTFPAVLQSSGLYSLSSVVTVPSSSLGTQTYICNVNHKPSNTKVDKKVEPKSCDKTHTCPPCPAPEAAGGPSVFLFPPKPKDTLMISRTPEVTCVVVDVSHEDPEVKFNWYVDGVEVHNAKTKPREEQYNSTYRVVSVLTVLHQDWLNGKEYKCKVSNKALPAPIEKTISKAKGQPREPQVYTLPPSRDELTKNQVSLTCLVKGFYPSDIAVEWESNGQPENNYKTTPPVLDSDGSFFLYSRLTVDKSRWQQGNVFSCSVMHEALHNHYTQKSLSLSPGK | EIVLTQSPGTLSLSPGERATFSCRSSHSIRSRRVAWYQHKPGQAPRLVIHGVSNRASGISDRFSGSGSGTDFTLTITRVEPEDFALYYCQVYGASSYTFGQGTKLERKRTVAAPSVFIFPPSDEQLKSGTASVVCLLNNFYPREAKVQWKVDNALQSGNSQESVTEQDSKDSTYSLSSTLTLSKADYEKHKVYACEVTHQGLSSPVTKSFNRGEC |
| E-2/N | 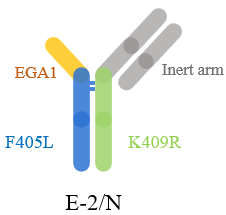 | EGA heavy chain construct  QVQLQESGGGLVQPGGSLRLSCAASGRTFSSYAMGWFRQAPGKQREFVAAIRWSGGYTYYTDSVKGRFTISRDNAKTTVYLQMNSLKPEDTAVYYCAATYLSSDYSRYALPQRPLDYDYWGQGTQVTVSSGGGGSDKTHTCPPCPAPELLGGPSVFLFPPKPKDTLMISRTPEVTCVVVDVSHEDPEVKFNWYVDGVEVHNAKTKPREEQYNSTYRVVSVLTVLHQDWLNGKEYKCKVSNKALPAPIEKTISKAKGQPREPQVYTLPPSREEMTKNQVSLTCLVKGFYPSDIAVEWESNGQPENNYKTTPPVLDSDGSFLLYSKLTVDKSRWQQGNVFSCSVMHEALHNHYTQKSLSLSPGK  Inert arm heavy chain construct  QVQLVQSGAEVKKPGASVKVSCQASGYRFSNFVIHWVRQAPGQRFEWMGWINPYNGNKEFSAKFQDRVTFTADTSANTAYMELRSLRSADTAVYYCARVGPYSWDDSPQDNYYMDVWGKGTTVIVSSASTKGPSVFPLAPSSKSTSGGTAALGCLVKDYFPEPVTVSWNSGALTSGVHTFPAVLQSSGLYSLSSVVTVPSSSLGTQTYICNVNHKPSNTKVDKKVEPKSCDKTHTCPPCPAPEAAGGPSVFLFPPKPKDTLMISRTPEVTCVVVDVSHEDPEVKFNWYVDGVEVHNAKTKPREEQYNSTYRVVSVLTVLHQDWLNGKEYKCKVSNKALPAPIEKTISKAKGQPREPQVYTLPPSRDELTKNQVSLTCLVKGFYPSDIAVEWESNGQPENNYKTTPPVLDSDGSFFLYSRLTVDKSRWQQGNVFSCSVMHEALHNHYTQKSLSLSPGK | EIVLTQSPGTLSLSPGERATFSCRSSHSIRSRRVAWYQHKPGQAPRLVIHGVSNRASGISDRFSGSGSGTDFTLTITRVEPEDFALYYCQVYGASSYTFGQGTKLERKRTVAAPSVFIFPPSDEQLKSGTASVVCLLNNFYPREAKVQWKVDNALQSGNSQESVTEQDSKDSTYSLSSTLTLSKADYEKHKVYACEVTHQGLSSPVTKSFNRGEC |
| E-12/N | 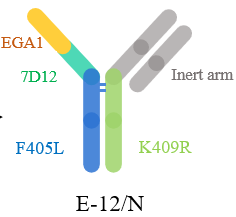 | EGA1 7D12 heavy chain construct  QVQLQESGGGLVQPGGSLRLSCAASGRTFSSYAMGWFRQAPGKQREFVAAIRWSGGYTYYTDSVKGRFTISRDNAKTTVYLQMNSLKPEDTAVYYCAATYLSSDYSRYALPQRPLDYDYWGQGTQVTVSS**GGGGSGGGGS**QVKLEESGGGSVQTGGSLRLTCAASGRTSRSYGMGWFRQAPGKEREFVSGISWRGDSTGYADSVKGRFTISRDNAKNTVDLQMNSLKPEDTAIYYCAAAAGWAWYGTLYEYDYWGEGTQVTVSSGGGGSDKTHTCPPCPAPELLGGPSVFLFPPKPKDTLMISRTPEVTCVVVDVSHEDPEVKFNWYVDGVEVHNAKTKPREEQYNSTYRVVSVLTVLHQDWLNGKEYKCKVSNKALPAPIEKTISKAKGQPREPQVYTLPPSREEMTKNQVSLTCLVKGFYPSDIAVEWESNGQPENNYKTTPPVLDSDGSFLLYSKLTVDKSRWQQGNVFSCSVMHEALHNHYTQKSLSLSPGK  Inert arm heavy chain construct  QVQLVQSGAEVKKPGASVKVSCQASGYRFSNFVIHWVRQAPGQRFEWMGWINPYNGNKEFSAKFQDRVTFTADTSANTAYMELRSLRSADTAVYYCARVGPYSWDDSPQDNYYMDVWGKGTTVIVSSASTKGPSVFPLAPSSKSTSGGTAALGCLVKDYFPEPVTVSWNSGALTSGVHTFPAVLQSSGLYSLSSVVTVPSSSLGTQTYICNVNHKPSNTKVDKKVEPKSCDKTHTCPPCPAPEAAGGPSVFLFPPKPKDTLMISRTPEVTCVVVDVSHEDPEVKFNWYVDGVEVHNAKTKPREEQYNSTYRVVSVLTVLHQDWLNGKEYKCKVSNKALPAPIEKTISKAKGQPREPQVYTLPPSRDELTKNQVSLTCLVKGFYPSDIAVEWESNGQPENNYKTTPPVLDSDGSFFLYSRLTVDKSRWQQGNVFSCSVMHEALHNHYTQKSLSLSPGK | EIVLTQSPGTLSLSPGERATFSCRSSHSIRSRRVAWYQHKPGQAPRLVIHGVSNRASGISDRFSGSGSGTDFTLTITRVEPEDFALYYCQVYGASSYTFGQGTKLERKRTVAAPSVFIFPPSDEQLKSGTASVVCLLNNFYPREAKVQWKVDNALQSGNSQESVTEQDSKDSTYSLSSTLTLSKADYEKHKVYACEVTHQGLSSPVTKSFNRGEC |

**Table S2:** The SEC-HPLC purity of monoclonal antibody and double antibody was detected by using an Agilent Infinity HPLC 1260 using detection at 220 nm absorbance.

| Ab ID | SEC-HPLC Purity (A220 nm) |
| --- | --- |
| E-1 | 96.99 % |
| E-1/N | 94.00 % |
| E-2 | 99.08 % |
| E-2/N | 92.36 % |
| E-12 | 96.13 % |
| E-12/N | 97.54 % |

**Table S3: EGFR density on different cell lines**

The antigen density of EGFR was detected by flow cytometry. The suspension was prepared by mixing 50 μL buffer containing 5 × 10^4^ cells with 50 μL buffer containing diluted PE-labeled Antibody and incubated in a refrigerator at 4°C in the dark for 0.5-1 h. The standard curve was prepared by using the BD QuantiBRITE^TM^ antigen PE Phycoerythrin Fluorescence Quantitation Kit.

| **Cell Name** | **EGFR Density** |
| --- | --- |
| RKO | 146963 |
| SNU-5 | 23703 |
| HCC827 | 1505288 |
| NCI-H1975 | 119807 |
| BxPC-3 | 19641 |
| HT-29 | 74439 |
| NCI-N87 | 44071 |
| Capan-2 | 29302 |
| Hs 578T | 54242 |
| MDA-MB-231 | 2483 |
| NCI-H196 | 64711 |

**Table S4:** **Results from the cell binding and ADCC reporter assays**

Eight dilutions of antibody were used from the initial concentration in the cell binding assay and the ADCC reporter assay. The following table contained test article, cell line, EC_50_ and Span values of the cell binding and ADCC Reporter Assay. The values were calculated by the Graphpad pseudo-cooperative diagram.

| Antibody | Cell line | Cell Binding | | ADCC Reporter Assay | |
| --- | --- | --- | --- | --- | --- |
|  |  | Binding EC_50_ (nM) | Binding Span  (gMFI of AF647) | ADCC EC_50_ (nM) | ADCC Span  (luminescence) |
| Inert Mab | RKO | 0.001113 | -35.59 | ~ 40686 | ~ 217034 |
| E-1/N | RKO | 1.12 | 10609 | 3.393 | 556.8 |
| E-1 | RKO | 0.02221 | 7818 | 0.3892 | 1561 |
| E-12/N | RKO | 0.09942 | 15417 | 0.1099 | 5170 |
| E-2/N | RKO | 0.1108 | 12413 | ~ 50526 | ~ 324255 |
| E-2 | RKO | 0.03808 | 9107 | 5.571 | 492.3 |
| Inert Mab | Capan-2 | 0.001954 | 53.88 | 1.979 | -24.48 |
| E-1/N | Capan-2 | 1.247 | 102527 | 2.288 | 29606 |
| E-1 | Capan-2 | 0.1643 | 74456 | 0.1719 | 27238 |
| E-12/N | Capan-2 | 0.4613 | 122864 | 0.06632 | 46576 |
| E-2/N | Capan-2 | 0.463 | 116525 | 0.9851 | 15054 |
| E-2 | Capan-2 | 0.2069 | 77505 | 0.2916 | 5763 |
| Inert Mab | NCI-N87 | 0.02277 | 23.32 | 4052834 | -10677264 |
| E-1/N | NCI-N87 | 1.568 | 114764 | 4.151 | 9854 |
| E-1 | NCI-N87 | 0.2987 | 83789 | 1.349 | 16358 |
| E-12/N | NCI-N87 | 0.8068 | 131594 | 0.07575 | 42979 |
| E-2/N | NCI-N87 | 0.9151 | 135799 | 1.476 | 5048 |
| E-2 | NCI-N87 | 0.4183 | 84368 | 1.782 | 2604 |
| Inert Mab | NCI-H196 | 0.01223 | 12.75 | 0.3325 | 10.24 |
| E-1/N | NCI-H196 | 0.7517 | 25764 | 4.133 | 3871 |
| E-1 | NCI-H196 | 0.1269 | 18865 | 4.979 | 9671 |
| E-12/N | NCI-H196 | 0.3658 | 31007 | 0.2491 | 10262 |
| E-2/N | NCI-H196 | 0.3205 | 28700 | 0.9735 | 384.3 |
| E-2 | NCI-H196 | 0.1779 | 19069 | ~ 79797 | ~ 2323100 |
| Inert Mab | BxPC-3 | ~ 8579 | ~ 16845 | 0.08915 | 126.6 |
| E-1/N | BxPC-3 | 1.616 | 492173 | 6.524 | 278555 |
| E-1 | BxPC-3 | 0.3932 | 340345 | 0.1276 | 179341 |
| E-12/N | BxPC-3 | 1.05 | 506248 | 0.08101 | 165139 |
| E-2/N | BxPC-3 | 0.9583 | 491755 | 0.5976 | 102513 |
| E-2 | BxPC-3 | 0.4836 | 337323 | 0.1442 | 66216 |
| Inert Mab | MDA-MB-231 | ~ 76581 | ~ 126560 | 1.821 | 82.81 |
| E-1/N | MDA-MB-231 | 0.3798 | 344351 | 1.262 | 111710 |
| E-1 | MDA-MB-231 | 0.07821 | 328166 | 0.02824 | 121044 |
| E-12/N | MDA-MB-231 | 0.3315 | 468514 | 0.02459 | 188168 |
| E-2/N | MDA-MB-231 | 0.3135 | 401851 | 0.4252 | 96447 |
| E-2 | MDA-MB-231 | 0.1269 | 359783 | 0.173 | 57043 |
| Inert Mab | NCI-H1975 | 2.813 | -11.22 | 1.144 | 57.44 |
| E-1/N | NCI-H1975 | 2.84 | 70355 | 4.657 | 54673 |
| E-1 | NCI-H1975 | 0.5311 | 48094 | 0.1307 | 36480 |
| E-12/N | NCI-H1975 | 1.448 | 70739 | 0.07053 | 92302 |
| E-2/N | NCI-H1975 | 1.527 | 72347 | 0.8863 | 8514 |
| E-2 | NCI-H1975 | 0.7051 | 48560 | 4.117 | 7519 |
| Inert Mab | SNU-5 | 0.003739 | 46.28 | 2.452E-09 | 6267597 |
| E-1/N | SNU-5 | 0.6371 | 93855 | 14.21 | 13760 |
| E-1 | SNU-5 | 0.2501 | 74123 | 0.8149 | 8629 |
| E-12/N | SNU-5 | 0.6001 | 115163 | 0.1728 | 20513 |
| E-2/N | SNU-5 | 0.5431 | 105470 | 1.163 | 1739 |
| E-2 | SNU-5 | 0.2642 | 70994 | 2.905 | 633.6 |
| Inert Mab | Hs 578T | 0.07937 | 34.4 | 0.1872 | 22.24 |
| E-1/N | Hs 578T | 0.9123 | 97298 | 10.06 | 21623 |
| E-1 | Hs 578T | 0.07691 | 68394 | 1.69 | 30240 |
| E-12/N | Hs 578T | 0.3087 | 113443 | 0.05793 | 31774 |
| E-2/N | Hs 578T | 0.2984 | 99065 | 0.7762 | 1925 |
| E-2 | Hs 578T | 0.1185 | 70353 | 23.57 | 3576 |
| Inert Mab | HCC827 | 32.9 | 227.9 | 0.1036 | 36.54 |
| E-1/N | HCC827 | 1.501 | 862937 | 3.956 | 89745 |
| E-1 | HCC827 | 0.3963 | 568710 | 0.2345 | 80827 |
| E-12/N | HCC827 | 1.171 | 848706 | 0.1387 | 84742 |
| E-2/N | HCC827 | 1.093 | 884481 | 0.4642 | 61846 |
| E-2 | HCC827 | 0.6957 | 582784 | 0.1091 | 39105 |
| Inert Mab | HT-29 | 0.0458 | 31.03 | 0.08728 | -3.28 |
| E-1/N | HT-29 | 1.067 | 33656 | 2.243 | 6047 |
| E-1 | HT-29 | 0.0483 | 40270 | 1.149 | 16198 |
| E-12/N | HT-29 | 0.1531 | 70644 | 0.04875 | 45397 |
| E-2/N | HT-29 | 0.1821 | 55623 | 0.6163 | 2556 |
| E-2 | HT-29 | 0.06311 | 43320 | 1.73 | 1679 |

**Table S5:** The affinity of antibody binding to human CD16a was determined by using the Octet (ForteBio). The biotinylated human CD16a was loaded to SA sensor. The test antibodies were prepared at a 2-fold dilution starting from 600 nM. The association and dissociation measurement were conducted in a kinetic buffer (PBS, pH 7.2, with 0.05% (w/v) Tween-20, 0.1% (w/v) BSA)

| Sample | KD (M) | ka (1/Ms) | kdis (1/s) | KD Error | ka Error | kdis Error |
| --- | --- | --- | --- | --- | --- | --- |
| E-1 | 2.870E-08 | 1.142E05 | 3.277E-03 | 8.184E-10 | 2.880E03 | 4.364E-05 |
| E-2 | 5.354E-08 | 1.076E05 | 5.758E-03 | 1.859E-09 | 3.484E03 | 7.185E-05 |
| E-12 | 9.128E-08 | 1.332E05 | 1.216E-02 | 2.524E-09 | 3.513E03 | 1.015E-04 |
| N | 5.592E-08 | 8.353E04 | 4.671E-03 | 1.626E-09 | 2.222E03 | 5.483E-05 |
| E-1/N | 7.088E-08 | 7.591E04 | 5.381E-03 | 2.058E-09 | 2.042E03 | 5.889E-05 |
| E-2/N | 1.115E-07 | 8.948E04 | 9.980E-03 | 4.238E-09 | 3.224E03 | 1.204E-04 |
| E-12/N | 5.367E-08 | 1.054E05 | 5.656E-03 | 1.642E-09 | 3.005E03 | 6.266E-05 |

**Figure S1**


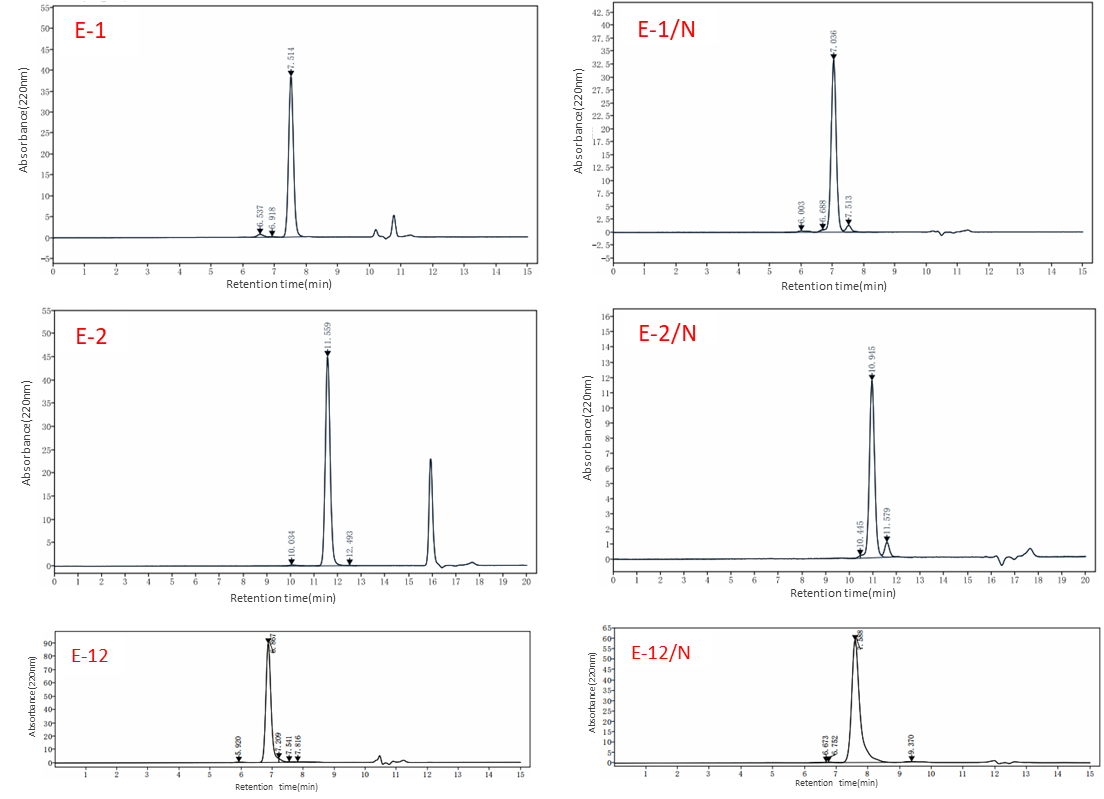


**Figure S1.** **Monodispersity of antibodies.** The SEC-HPLC purity of monoclonal antibodies and double antibodies were detected using Agilent AdvanceBio SEC column 300Å and running with 150 mM KPi pH 7.3 by Agilent Infinity HPLC 1260 using detection at 220 nm absorbance.

**
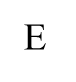

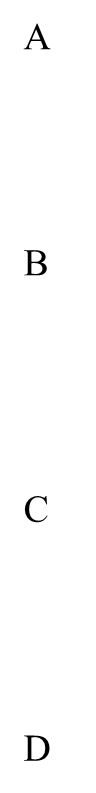
Figure S2**

**
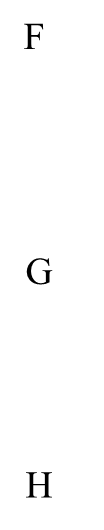
**

**Figure S2. Cell binding and ADCC reporter assays of monovalent and bivalent antibodies.** Bivalent antibodies E-1 and E-2 were compared to monovalent antibodies E-1/N and E-2/N by cell binding and ADCC reporter assays. The inert antibody was used as a vehicle control. The cell lines of triple negative breast cancer (A), colon cancer (B), gastric cancer (C and D), pancreatic cancer (E and F) and lung cancer (G and H) were tested. For the cell binding assays (left part of all figures), the X axes were antibody concentrations, and the Y axes were geometric mean fluorescence intensity (gMFI) of antibody bound to the cell surface. For the ADCC reporter assays (right part of all figures), the X axes were antibody concentrations, and the Y axes were luminescence values.

**Figure S3**

**
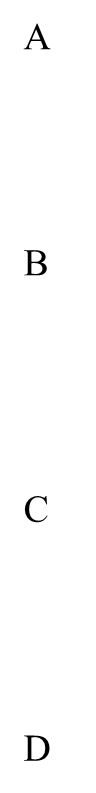
**

**
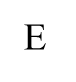
**

**
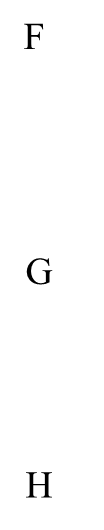
**

**Figure S3. Cell binding and ADCC reporter of single paratopic and biparatopic antibodies.** Biparatopic E-12/N was compared to single paratopic monovalent antibodies E-1/N and E-2/N by cell binding and ADCC reporter assays. An inert antibody was taken as vehicle control. Cell lines of triple negative breast cancer (A), colon cancer (B), gastric cancer (C and D), pancreatic cancer (E and F) and lung cancer (G and H) were tested. For cell binding (left part of all figures), the X axes were antibody concentrations, and the Y axes were geometric mean fluorescence intensity (gMFI) of antibody bound to the cell surface. For ADCC reporter assay (right part of all figures), the X axes were antibody concentrations, and the Y axes were luminescence values.

**Figure S4**

**
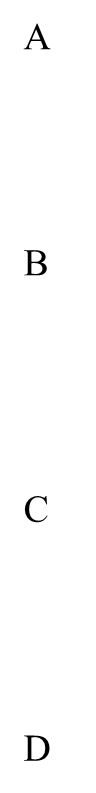

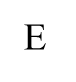
**

**
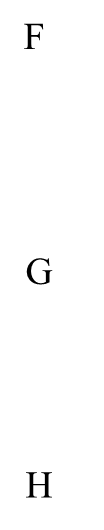
**

**Figure S4. Cell binding and ADCC reporter of biparatopic and bivalent antibodies.** The biparatopic antibody E-12/N was compared to bivalent antibodies E-1 and E-2 by cell binding and ADCC reporter assays. An inert antibody was taken as vehicle control. The cell lines of triple negative breast cancer (A), colon cancer (B), gastric cancer (C and D), pancreatic cancer (E and F) and lung cancer (G and H) were tested. For the cell binding assays (left part of all figures), the X axes were antibody concentrations, and the Y axes were geometric mean fluorescence intensity (gMFI) of antibody bound to the cell surface. For the ADCC reporter assays (right part of all figures), the X axes were antibody concentrations, and the Y axes were luminescence values.
